# Atrial Fibrillation: Biophysics, Molecular Mechanisms, and Novel Therapies

**DOI:** 10.1155/2015/780671

**Published:** 2015-07-09

**Authors:** Alexey V. Glukhov, Leonid V. Rosenshtraukh, Anamika Bhargava, Michele Miragoli, Bas J. D. Boukens

**Affiliations:** ^1^National Heart and Lung Institute, Imperial College, London W12 0NN, UK; ^2^Institute of Experimental Cardiology, Cardiology Research Centre, Moscow 121552, Russia; ^3^Department of Biotechnology, Indian Institute of Technology Hyderabad, Telangana 502205, India; ^4^Humanitas Clinical and Research Center, 20090 Rozzano, Italy; ^5^Department of Biomedical Engineering, George Washington University, Washington, DC 63119, USA; ^6^Department of Anatomy, Embryology and Physiology, University of Amsterdam, 1105 AZ Amsterdam, Netherlands

Atrial fibrillation (AF) affects millions of people worldwide and is associated with increased morbidity and mortality [1]. The incidence of AF is expected to rise with aging of the population. Research over the past decades has identified a multitude of pathophysiological processes contributing to the initiation, maintenance, and progression of AF. Therefore, a comprehensive understanding of AF pathophysiology is needed to foster the development of improved diagnostic, pharmacological, and nonpharmacological therapeutic approaches to improve clinical management. The focus of this special issue of this journal is to capture most recent advances in the study of AF with the aim of directing further research.

Conventional mechanisms linked to AF are diverse and expertly reviewed in various manuscripts [2, 3]. However, progression in the field of AF research may come from an unconventional view-angle. For example, M. Miragoli and A. V. Glukhov reviewed the role of myofibroblasts as novel targets for cardiac arrhythmias with the aim of describing and evaluating the implications of noncardiomyocyte view in the context of AF. B. Weil and C. Ozcan discussed the pathophysiological remodelling in AF in comparison with that occurring in hibernating myocardium, attempting to identify common molecular mechanisms and proposing possible future therapeutic implications of this emerging paradigm.

In addition, the issue also contains the study of  F. Cacciani and M. Zaniboni who used a computational approach to study the source-sink relationship between the sinoatrial node and surrounding atrium in control conditions and under different simulated chronotropic interventions. The authors defined a safety-factor for pacemaker-to-atrial action potential conduction and tested the effects of different antiarrhythmic-like interventions. Analytical study of C. Loardi et al. found that there is an association between postoperative left atrium dimensions and “kick” restoring and an influence of right atrium contraction on left ventricle function in patients that underwent radiofrequency (Maze) procedure. Their study also highlights the deleterious influence of arrhythmia “chronic state” on procedural success and importance of the cardiac right side, an entity somehow overlooked.

The initial presentation of undiagnosed AF is often a stroke. Therefore, methods for early detection of AF are highly demanded. In this special issue, M. D. Zink et al. investigated whether heart beat cycle length measurement assessed by a novel algorithm for a ballistocardiography (BCG) foil is feasible during sinus rhythm and AF. The latter might offer new possibilities for noninvasive heart rate and thereby AF monitoring. Another approach to detect AF would be the use of biomarkers. P. J. Howlett et al. summarized the available evidence for electrophysiological, molecular, and morphological biomarkers to improve the detection of paroxysmal AF with reference to the underlying mechanisms for the conduction. F.-C. Su et al. performed a meta-analysis to investigate whether serum level of high-sensitivity C-reactive protein (hs-CRP), a protein produced by the liver during infection, tissue injury, and chronic inflammation, can predict the efficacy of AF treatment with atorvastatin.

The coagulative state in patients with chronic AF is higher than that in patients with sinus rhythm [4]. As a result, the incidence of thromboembolism is significantly higher in patients with AF [5] which makes the latter an important risk factor of cerebral embolism. In this issue, F. Shamoun et al. reviewed the new generation of anticoagulants, along with their efficacy and safety data. Their study suggests that the novel oral anticoagulants indeed can reduce the relative incidence of stroke in patients with AF. Similarly, A. Panisello-Tafalla et al. examined the effectiveness of the use of oral anticoagulation medication, recommended by national guidelines for stroke preventions. They suggest that the low efficiency of oral anticoagulants results from the association between their underuse and undiagnosed AF. The treatment of platelet aggregation in patients with AF requires a specific approach as highlighted by A. M. Martischnig et al. who found that dabigatran as compared to phenprocoumon had no impact on adenosine diphosphate (ADP) induced platelet aggregation in AF patients neither with nor without concomitant clopidogrel therapy.

The extensive work reviewed in this special issue highlights the enormous advances achieved in understanding AF pathophysiology over the past 20 years and suggests an importance of the mechanism-based approaches for therapeutic breakthroughs. We believe that the future of AF management will be determined by the application of new scientific insights into the underlying mechanistic determinants.
